# Lysosomal Calcium Channels in Autophagy and Cancer

**DOI:** 10.3390/cancers13061299

**Published:** 2021-03-15

**Authors:** Yi Wu, Peng Huang, Xian-Ping Dong

**Affiliations:** 1 Collaborative Innovation Center for Biomedicine, School of Clinical Medicine, Shanghai University of Medicine and Health Sciences, Shanghai 201318, China; Wuy_20@sumhs.edu.cn; 2 Departments of Physiology and Biophysics, Dalhousie University, 5850 College Street, Halifax, NS B3H 4R2, Canada

**Keywords:** lysosome, ion channel, calcium, autophagy, cancer

## Abstract

**Simple Summary:**

Autophagy is a cellular self-eating process that uses lysosome, the waste disposal system of the cell, to degrade and recycle intracellular materials to maintain cellular homeostasis. Defects in autophagy are linked to a variety of pathological states, including cancer. Calcium is an important cellular messenger that regulates the survival of all animal cells. Alterations to calcium homoeostasis are associated with cancer. While it has long been considered as cellular recycling center, the lysosome is now widely known as an intracellular calcium store that regulates autophagy and cancer progression by releasing calcium via some ion channels residing in the lysosomal membrane. In this review, we summarize existing mechanisms of autophagy regulation by lysosomal calcium channels and their implications in cancer development. We hope to guide readers toward a more in-depth understanding of the importance of lysosomal calcium channels in cancer, and potentially facilitate the development of new therapeutics for some cancers.

**Abstract:**

Ca^2+^ is pivotal intracellular messenger that coordinates multiple cell functions such as fertilization, growth, differentiation, and viability. Intracellular Ca^2+^ signaling is regulated by both extracellular Ca^2+^ entry and Ca^2+^ release from intracellular stores. Apart from working as the cellular recycling center, the lysosome has been increasingly recognized as a significant intracellular Ca^2+^ store that provides Ca^2+^ to regulate many cellular processes. The lysosome also talks to other organelles by releasing and taking up Ca^2+^. In lysosomal Ca^2+^-dependent processes, autophagy is particularly important, because it has been implicated in many human diseases including cancer. This review will discuss the major components of lysosomal Ca^2+^ stores and their roles in autophagy and human cancer progression.

## 1. Introduction

The lysosome is an acidic single-membrane organelle first discovered in 1955 by Christian de Duve while investigating the mechanism of action of insulin [1,2,3]. It contains more than 60 hydrolytic enzymes (e.g., nucleases, glycosidase, phosphatases, sulfatases, lipases, and proteases), which are active at the luminal acidic environment (pH 4.5–5.0) established by the vacuolar-ATPase (V-ATPase) proton pump [4,5]. Since its discovery, the lysosome has mainly been considered to be the center of waste disposal, which digests unwanted macromolecules, damaged and senescent organelles, microbes, and other particles delivered via endocytosis, autophagy, and phagocytosis [4,6,7,8]. Once degraded, some breakdown products such as free fatty acids, amino acid, monosaccharides and nucleotides are transported back to the cytosol via specific exporters in the lysosome membrane for reutilization in anabolic processes [9,10]. Lysosomes also contain more than 60 membrane proteins that are implicated in the maintenance of the lumen homeostasis, especially ionic homeostasis and membrane potential, in the control of molecular export across the lysosomal membrane, and in lysosomal membrane trafficking (i.e., fusion and fission). The functions of lysosomes in material degradation, catabolite export, or trafficking are key to maintaining cellular homeostasis, the perturbations of which often lead to lysosomal storage diseases (LSDs) [11].

Recent studies have shown that the lysosome is not only the terminal degradative compartment, but also a multifunctional signaling hub that integrates the cell’s responses to nutrient status, growth factors, and hormones. Noticeably, in order to adapt to changes in cellular environment, the lysosome has a nutrient-sensing mechanism involving mammalian/mechanistic target of rapamycin complex 1 (mTORC1) and transcription factor EB (TFEB) [12,13,14]. mTORC1 is capable of sensing a myriad of nutrient and energy cues, phosphorylating numerous cell growth-related substrates including TFEB, and thus governing the balance between catabolic and anabolic metabolic pathways in the cell [15]. TFEB can bind to a palindromic 10 bp nucleotide motif, named the coordinated lysosomal expression, and regulation (CLEAR) element, and activate the transcription of many genes encoding lysosomal proteins and autophagy-related proteins [16,17]. Under nutrient sufficient conditions, TFEB is sequestered away from the nucleus due to being phosphorylated by mTORC1. Conversely, under starvation, TFEB becomes dephosphorylated due to a reduction in mTORC1 and an activation of calcineurin (CaN), a Ca^2+^ and calmodulin (CaM) dependent serine/threonine protein phosphatase, and translocates to the nucleus to promote the transcription of the CLEAR element, which subsequently promotes autophagy-lysosome pathway as well as exocytosis and phagocytosis [8,18,19,20].

Autophagy is an evolutionarily conserved cellular degradative process that is induced by nutrient and energy starvation. It is a fundamental cellular program for cells to maintain intracellular energy and nutrient homeostasis and to protect cells against stress. During autophagy, intracellular components such as macromolecules and unwanted organelles are engulfed in autophagosomes that then fuse with lysosomes to form autolysosomes for degradation [21]. Lysosomal Ca^2+^ plays a key role in autophagy. For example, Transient Receptor Potential Mucolipin 1 (TRPML1, encoded by MCOLN1 gene), an important Ca^2+^ channel in the lysosome, governs autophagy through regulating both mTORC1 [22,23] and TFEB [24,25]. Because autophagy has an essential role in cellular homeostasis, it is implicated in various physiological processes and human diseases. Among them, the roles of autophagy in cancer have been extensively studied. Thus, in this review we focus on the role of lysosomal Ca^2+^ channels in autophagy and cancer.

## 2. Lysosomal Ca^2+^ Homeostasis

**Figure 1:**
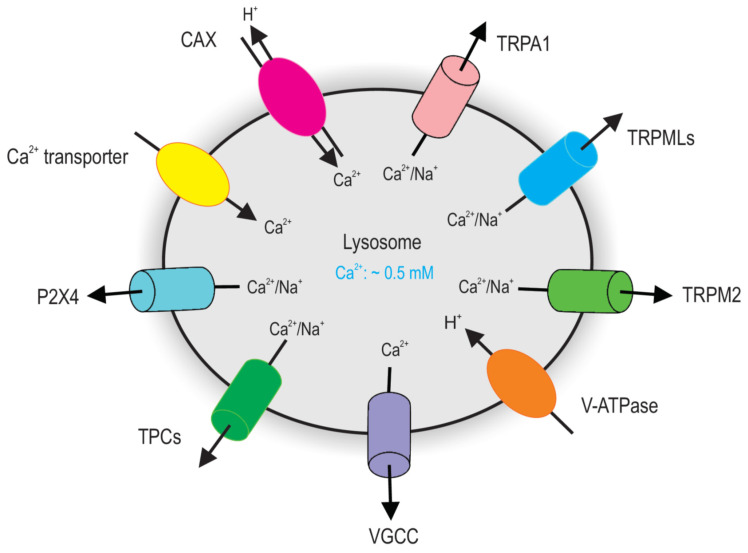
Major Ca^2+^ releasing channels and transporters on lysosomes. TRPML channels and TPC channels are major groups of Ca^2+^ channels on lysosomes that have been definitely defined by endolysosome-patch-clamp. Given the topology of the TRPML proteins at the endolysosomal membrane and the electrical properties of the endolysosome, TRPML opening leads to Ca^2+^ and Na^+^ release from the endolysosome to the cytosol. Activation of TPCs release lysosomal Na^+^ and Ca^2+^. Lysosomes accumulate Ca^2+^ using a putative Ca^2+^ Transporter or Ca^2+^/H^+^ exchanger (CAX).

The lysosome is a significant intracellular Ca^2+^ store, which coordinates cellular adaptive responses [12,15,26,27,28,29,30]. In the early 1990s, nicotinic acid adenine dinucleotide phosphate (NAADP) was discovered to be a potent mobilizer of Ca^2+^ from stores separated from those sensitive to inositol 1,4,5-trisphosphate (IP3) and cyclic ADP-ribose (cADPR) [31]. It was later shown that the NAADP-sensitive Ca^2+^ store is the functional equivalent of the lysosomal system, suggesting the lysosome may function as a Ca^2+^ store [32]. Indeed, in the same time, the lysosomal Ca^2+^ concentration was estimated to be ~0.5 mM, approximately 5000-fold higher than the cytosolic Ca^2+^ concentration (∼100 nM) [33,34,35]. This Ca^2+^ gradient across the lysosomal membrane is thought to be established by an unidentified Ca^2+^/H^+^ exchanger or Ca^2+^ transporter [36,37]. Multiple Ca^2+^ sensors including CaM, apoptosis linked gene 2 (ALG-2), and synaptotagmin 7 (Syt 7) associated with lysosomes have also been identified [22,33,38,39,40,41]. This further increases the diversity of lysosomal Ca^2+^-dependent processes. Recently, several groups have made new discoveries elucidating the molecular machinery underlying lysosomal Ca^2+^ release, Ca^2+^ signaling, and Ca^2+^ uptake (Figure 1).

### 2.1. Lysosomal Ca^2+^ Release

**Figure 2:**
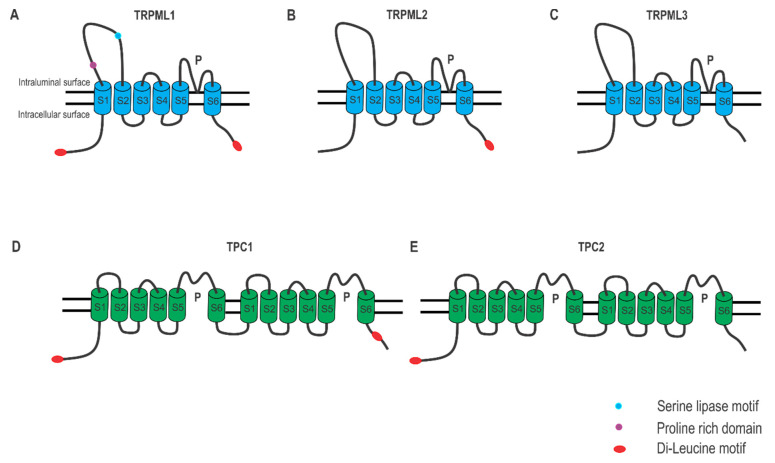
Structure of TRPML channels and TPC channels. (**A**–**C**) TRPML channels have a six membrane-spanning region (S1–S6), a putative pore region (P), and a large luminal loop between S1 and S2. Dileucine motifs in TRPML1 and TRPML2 at their C- and/or N- termini determine their intracellular endolysosomal localization. The endolysosomal localization of TRPML3 is determined by its heteromultimerization with other TRPMLs. Functional TRPMLs are tetramers. (**D**,**E**) TPC Channels are comprised two repeated domains, with each of them containing six transmembrane helices (S1–S6) and a pore loop (P) domain. TPC1 has two di-leucine motifs, while TPC2 has only one.

Lysosomal Ca^2+^ is mainly released through TRPML1, which belongs to the TRPML channel family including TRPML1, TRPML2, and TRPML3. TRPML proteins form tetramers, and each pore-forming subunit contains six transmembrane domains (TM1–TM6 or S1–S6) (Figure 2A–C). While TRPML1 is predominantly localized on the late endosomes and the lysosomes, TRPML2 and TRPML3 are primarily on the recycling endosomes and the early endosomes, respectively. In contrast to TRPML1, which is ubiquitously expressed in all tissues, TRPML2 and TRPML3 expressed in specific organs [42].

TRPML1 is a Ca^2+^-permeable, non-selective cation channel that can be activated by phosphatidylinositol 3,5-bisphosphate [PI(3,5)P2] [43,44] but inhibited by mTORC1 [22,45]. Normally under nutrient rich conditions, mTORC1 phosphorylates and inhibits TRPML1. During starvation, a reduction in mTORC1 activates TRPML1, stimulating the TFEB-dependent autophagy pathway, helping the cell survive extreme conditions [22]. TRPML1 is also regulated by some compounds, including mucolipin-specific synthetic agonists (ML-SAs) [46,47] and synthetic inhibitors ML-SIs [45] (Table 1). Physiologically, TRPML1 plays an important role in membrane trafficking, autophagy, lysosomal biogenesis, and lysosomal exocytosis [42,48]. Deficient TRPML1 causes type IV mucolipidosis (ML-IV), an autosomal recessive lysosomal storage disorder showing psychomotor retardation. Impaired TRPML1 has also been implicated in several other LSDs [49].

**Table 1:** Agonists and antagonists of TRPMLs and TPCs.

Lysosomal Ca^2+^ Channels	Agonists	Antagonists
TRPML1	ML-SAs [46,47,50,51,52] SF-51 [50] MK6-83 [53]	ML-SIs [45,47,51,52,54,55]
TRPML2	ML-SAs ML2-SA1 [56]	ML-SIs
TRPML3	ML-SAs SFs [50]	ML-SIs
TPC1	LyNa-VA and LyNA [57]	Tetrandrine [58,59] Ned-19 [60]
TPC2	TPC2-A1-N and TPC2-A1-P [61] LyNa-VA and LyNA [57]	Tetrandrine [58,59] Ned-19 [60]

**Figure 3:**
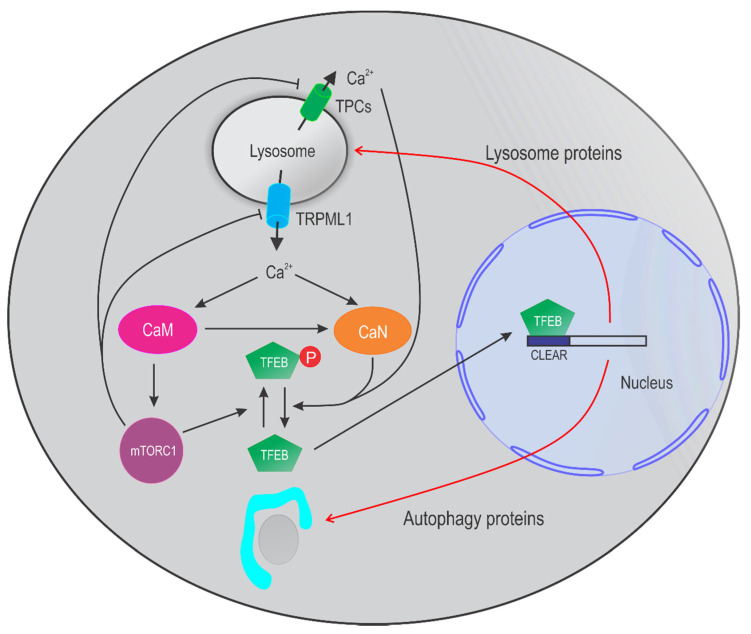
Lysosomal Ca^2+^ signaling pathways that regulate autophagy and lysosome biogenesis. In nutrient rich condition, mTORC1 phosphorylates and inhibits TRPML1, TPC2, and TFEB. Nutrient starvation activates TRPML1 due to a reduction of mTORC1 activity. Ca^2+^ released via TRPML1 activates calmodulin (CaM). On the one hand, activated CaM induces calcineurin (CaN) activation and subsequent TFEB dephosphorylation. Dephosphorylated TFEB translocates to nucleus to activate the CLEAR genes to promote lysosomal biogenesis and autophagy. On the other hand, CaM stimulates mTORC1 for basal protein synthesis, preventing cell death. Therefore, TRPML1/CaM coordinates CaN and mTORC1 to maintain homeostasis during nutrient starvation. TPCs may also participate in TFEB regulation in some cell types.

Two-pore channels, including TPC1 and TPC2 in human, are widely expressed in the endolysosomal system. They form a homodimer, with each pore-forming subunit comprising two repeats of six-transmembrane domains (Figure 2D,E). Functional TPC1/2 are dimetric non-selective Na^+^/Ca^2+^ channels that can be evoked by both NAADP and PI(3,5)P2 [43,57,62,63,64]. While TPC1 is expressed in both the early endosomes and the lysosomes, TPC2 is predominantly present on the lysosomal membranes [65]. TPCs are normally suppressed by intracellular ATP through mTORC1 kinase. Starvation and pharmacological inhibition of mTORC1 leads to TPC-dependent Ca^2+^ release to regulate autophagy [65,66] (Figure 3). Pharmacologically, both TPC1 and TPC2 are activated by a group of antidepressants LyNa-VAs and LyNA (i.e., Riluzole) [57]. TPC2 is also activated by two lipophilic and structurally distinct compounds TPC2-A1-N and TPC2-A1-P [61]. On the other hand, a structural analog of NAADP Ned 19 [60] and a bis-benzylisoquinoline alkaloid Tetrandrine [58,59] have been suggested to inhibit both TPC1 and TPC2, although definitive evidence supporting this claim is still lacking (Table 1).

P2X4 is a trimeric 2 helix-transmembrane channel that belongs to the ionotropic P2X-family ATP receptors. P2X4 is gated by intraluminal ATP to mediate Ca^2+^ release from the lysosome. P2X4 activity is also regulated by lysosomal pH with acidic luminal pH suppressing its activity [67,68]. Activation of P2X4-dependent Ca^2+^ release has been reported to promote lysosome fusion in a CaM-dependent manner [38].

Several other Ca^2+^ channels have been suggested to be expressed in the lysosome as well. These include transient receptor potential ankyrin 1 (TRPA1), a Ca^2+^-permeable non-selective cation channel found in somatosensory neurons [69]; transient receptor potential melastatin 2 (TRPM2), a Ca^2+^ permeable non-selective cation channel gated by ADP-ribose and Ca^2+^ [70]; snd P/Q-type voltage gated Ca^2+^ channels (VGCCs), Ca^2+^ channels regulating the fusion of autophagosomes with lysosomes in neurons [64]. However, their functions in lysosomes have not been determined by direct lysosome-patch-clamp recording. Currently, the major Ca^2+^ channels involved in the autophagy are TRPMLs and TPCs channels.

### 2.2. Lysosomal Ca^2+^ Store Refilling

Due to the storage capacity, the lysosome needs to be refilled with Ca^2+^ after release. The refilling of lysosomal Ca^2+^ store seems to be dependent on the lysosomal H^+^ (Figure 1), because (1) elevation of lysosomal pH and inhibition of V-ATPase deplete Ca^2+^ store [33,36,46]; (2) Ca^2+^ store is maintained through the Ca^2+^-H^+^ exchanger (CAX) in the vacuoles of yeast and plant, which is from some respects the equivalent of lysosomes in animal cells [36]. Therefore, it is conceivable that Ca^2+^/H^+^ exchanger may take part in the Ca^2+^ uptake in the lysosome [33,36]. However, in addition to triggering lysosomal Ca^2+^ release, manipulation of lysosomal pH may affect lysosomal Ca^2+^ concentration or its measurement. For example, because the lysosome contains substantial Ca^2+^ buffers [71] that binds Ca^2+^ much better at neutral pH [72], increasing lysosomal pH may reduce lysosomal free Ca^2+^ without necessarily triggering lysosomal Ca^2+^ release and affecting total Ca^2+^ content [72]. Lysosomal pH may also affect the chromophore fluorescence and Ca^2+^-binding affinity (*K*_d_) of Ca^2+^ dyes sequestered in the lumen, because *K*_d_ drops significantly when lysosomal pH increases [71]. Additionally, lysosomal pH elevation may indirectly affect lysosomal Ca^2+^ homeostasis by regulating membrane fusion and fission between compartments containing different amounts of Ca^2+^, H^+^, and their buffers [36,73]. To study lysosomal Ca^2+^ refilling under more physiological conditions, Garrity et al. [73] have adopted a lysosome-targeted GCaMP (fused to the cytosolic N-terminus of TRPML1) [46] to directly monitor the real-time changes in lysosomal Ca^2+^. They reported that inhibition of the H^+^ gradient in the lysosome, for example, by V-ATPase inhibitors, did not affect Ca^2+^ refilling. Therefore, the identity of the channel or exchanger mediating lysosomal Ca^2+^ uptake is still unclear [33,36]. In line with this, it has been previously suggested that Ca^2+^ uptake into lysosomes is mediated by a low-affinity Ca^2+^ transporter [74]. Very recently, the P5 ATPase ATP13A2 was suggested to mediate lysosomal Ca^2+^ entry [37]. However, whether ATP13A2 transport Ca^2+^ into lysosomes is regulated by H^+^ gradient across the lysosomal membrane remains unclear.

ER Ca^2+^ has been suggested to play an important role in Ca^2+^ refilling [73]. Moreover, it is IP3 receptors (IP3Rs) rather than ryanodine receptors (RyRs) that are involved in this process. Thus, a three step model has been proposed based on the structurally intimate localization of the ER and the lysosome [75,76]: (1) refilling stimulated by increased peri-lysosomal Ca^2+^ and/or decreased lysosomal Ca^2+^; (2) formation of ER-lysosome membrane contact sites (MCS) [73,77,78,79]; and (3) Ca^2+^ transport from the ER to the lysosome through functional ER–lysosome membrane contact sites.

### 2.3. Crosstalk between Lysosomes and Other Ca^2+^ Stores

**Figure 4:**
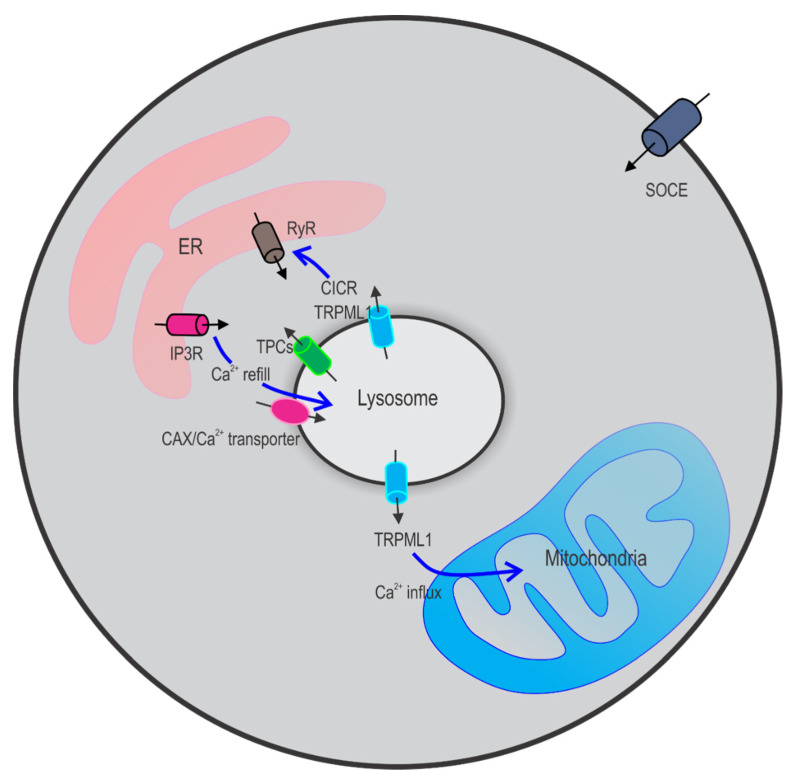
Interaction between lysosomes and the endoplasmic reticulum (ER) and mitochondria. On the one hand, lysosomal Ca^2+^ release through ion channels such as TRPML1 and TPC2 triggers Ca^2+^ release from the ER Ca^2+^ stores. On the other hand, when lysosomal Ca^2+^ store is depleted, Ca^2+^ released via IP3R from ER refills lysosomes. Lysosomal TRPML1 also promotes Ca^2+^ transfer from lysosomes to mitochondria. CICR: calcium induced calcium release; SOCE: store operated calcium entry.

By releasing and taking up Ca^2+^, the lysosomes, along with the ER, modulate cytosolic Ca^2+^ signaling events. On the one hand, lysosomal Ca^2+^ release can induce Ca^2+^ release from the ER using a mechanism similar to the Ca^2+^ induced Ca^2+^ release (CICR). This further causes Ca^2+^ entry from the extracellular space due to the depletion of the ER Ca^2+^ store, evoking global Ca^2+^ signals in human cells [80]. Both TRPML1 [81] and TPC2 [82,83,84]-mediated Ca^2+^ release are involved in this process. On the other hand, increasing evidence has suggested that, when lysosomal Ca^2+^ store is depleted, Ca^2+^ in the ER is transported to the lysosome via IP3R to refill the lysosome with Ca^2+^ [73,85]. The bidirectional Ca^2+^ signaling between the lysosome and the ER [86] has also been supported by the identification of the ER–lysosome contact sites [77,87] (Figure 4). In addition, new evidence suggests that the lysosome may also act as a Ca^2+^ buffer to shape extracellular Ca^2+^ entry and the ER Ca^2+^ re-uptake [85].

Interorganellular contacts between mitochondria and lysosomes have also been suggested recently [88,89,90]. Using high-resolution microscopy, Wong et al. [88,89] identified a dynamic formation of contact sites between mitochondria and lysosomes that were regulated by Rab7, a small GTPase associated with lysosomes. These contact sites allow bidirectional crosstalk between mitochondria and lysosomes and regulate the organelle network dynamics, such as mitochondrial fission. By high spatial and temporal resolution live-cell microscopy, they further suggested a role of the mitochondria–lysosome contact in regulating mitochondrial Ca^2+^ dynamics by lysosomal TRPML1, i.e., in the presence of mitochondria–lysosome contact, lysosomal Ca^2+^ release through TRPML1 promotes Ca^2+^ transfer to mitochondria [90] (Figure 4). Taken together, emerging evidence suggests that the lysosome may regulate intracellular Ca^2+^ signaling by buffering ER Ca^2+^ release and uptake, mitochondrial Ca^2+^ release uptake, and extracellular Ca^2+^ influx.

## 3. Autophagy

Autophagy is a self-eating process that is important for balancing sources of energy at critical times in development and in response to nutrient stress. The cell uses an autophagy pathway to degrade and recycle cytoplasmic constituents such as protein aggregates, lipids, and complete organelles for cell survival. It is especially important in postmitotic cells, such as muscles and neurons, where accumulation of aggregated proteins and damaged organelles often results in cell death [91,92,93]. Indeed, suppression of autophagy causes compromised neuron and muscle differentiation [94,95,96] as well as neurodegeneration [91,92,93] and myofiber degeneration [97,98].

In most cells, autophagy is kept at a low level under nutrient rich condition. However, stressful conditions, such as nutritional deprivation, oxidative stress, Ca^2+^ overload, pathogen infection, and other diseases, activate autophagy. By upregulating autophagy under such conditions, cells degrade macromolecules into their building blocks for reutilization, thereby adapting to extreme conditions and maintaining cellular homeostasis.

There are three types of autophagy: macroautophagy [99,100], microautophagy [99,101], and chaperone-mediated autophagy (CMA) [102,103]. Macroautophagy is the most common form of autophagy, which is characterized by the formation of a typical double-membrane cistern (so called phagophore) that extends and engulfs part of the cytoplasm to form a whole vesicle (so called autophagosome). The autophagosome ultimately fuses with a lysosome to form an autolysosome [104,105,106]. In this review, we focus on macroautophagy (hereafter referred to as autophagy).

The process of autophagy is controlled by multiple complexes of proteins encoded by evolutionarily conserved, autophagy-related (ATG) genes, which were originally identified in yeast. The products of these ATG genes, together with other autophagy-related factors, regulate autophagosome formation, tethering, and fusion with lysosomes [107,108,109,110]. Autophagy is also regulated by some non-ATG proteins. For example, in the presence of nutrients, ATG1/ULK1 is phosphorylated by mTORC1 [111], thereby inhibiting autophagy initiation [112]. mTORC1 can also phosphorylate and inactivate TFEB, repressing autophagy [13,113]. In the absence of nutrients, TRPML1-metiated lysosomal Ca^2+^ release activates CaN, which further causes TFEB dephosphorylation and nuclear translocation, thereby promoting autophagy [25].

Studies of mammalian systems have highlighted many important roles of autophagy in health and diseases including cell growth [114] and differentiation [96], LSDs [49,115], neurodegenerative diseases [92,93], bacterial infections [116], and cancers [117,118].

## 4. Lysosomal Ca^2+^ in Autophagy

### 4.1. TRPML Channels in Autophagy

It is widely accepted that Ca^2+^ can regulate autophagy, while mechanisms differ depending on the conditions. The interplay between lysosomal Ca^2+^ signal and autophagy has also been reported. In line with this, several lysosomal Ca^2+^-permeable channels have been suggested to regulate autophagy [119,120,121].

As a key Ca^2+^ release channel in the lysosomal membrane, TRPML1 deficiency leads to defective autophagy including accumulation of autophagosomes and aggregation of p62 proteins [122,123,124,125]. Growing evidence suggests that TRPML1 plays multifaceted roles in autophagy. Under normal conditions, mTORC1 phosphorylates and inhibits both TRPML1 and TFEB. Cellular stress activates TRPML1 due to mTORC1 inhibition. This further activates downstream pathways including (1) CaM/CaMKKβ/AMPK-dependent autophagosome formation [126], (2) ALG-2-dependent lysosome centripetal movement to promote autophagosome–lysosome fusion [45], (3) proteolytic degradation in autolysosomes [127], (4) Syt7-dependent lysosomal exocytosis to remove cellular garbage [45,128], (5) CaM-dependent mTORC1 reactivation to prevent cell death by increasing protein synthesis and promote lysosome reformation [22,23], and (6) CaN/TFEB activation to continuously supply lysosome and autophagy proteins [24,25,47] (Figure 3). Because TRPML1 is also a target of TFEB, a positive feedback is established to largely potentiate autophagy during stress [24,25]. Thus, TRPML1 is involved in several steps of autophagy including autophagosome formation, autophagosome maturation, autolysosome degradation, and autophagic lysosome reformation.

In addition to TRPML1, TRPML3 also takes part in autophagy regulation. TRPML3 has been found in the plasma membrane and multiple intracellular compartments, including autophagosomes, early endosomes, late endosomes, and lysosomes. The multiple compartmental localization of TRPML3 suggests that TRPML3 is dynamically expressed in different compartments and plays a role in membrane trafficking. Indeed, TRPML3 is accumulated in the plasma membrane upon inhibition of endocytosis and is recruited to autophagosomes upon induction of autophagy, thereby regulating endocytosis and autophagy [129]. Specifically for autophagy, TRPML3 overexpression increases while its knock-down or expression of the channel-dead dominant negative TRPML3 reduces autophagy [129]. Mechanistically, emerging evidence suggests that palmitoylation at its C-terminal region is required for TRPML3′s function in autophagosome formation, potentially by controlling its trafficking to autophagic structures [130], where TRPML3 may promote autophagosome maturation by providing Ca^2+^ in the fusion process through a specific interaction with GATE16, a mammalian ATG8 homologue [131].

### 4.2. TPC Channels in Autophagy

The role of TPCs in autophagy has been conflicting. Pereira et al. [132] showed that in astrocytes, NAADP and TPC2 overexpression increased the levels of autophagy markers, LC3 and beclin-1, and NAADP-mediated increases in LC3II levels were reduced in cells expressing a dominant–negative TPC2 construct. In the meantime, Leucine-rich repeat kinase 2 (LRRK2), an important regulator of autophagy involved in late-onset familial Parkinson’s disease (PD) [133], activated the CaMKKβ)/AMPK pathway, which was followed by a persistent increase in autophagosome formation. These effects were mimicked by the lysosomal Ca^2+^-mobilizing messenger NAADP and reversed by an NAADP receptor antagonist or expression of dominant–negative receptor constructs, suggesting that TPC2-mediated lysosomal Ca^2+^ release may promote autophagy [134]. However, skeletal muscles from animals lacking TPC2 displayed an enhanced autophagy flux [135]. In addition, loss of TPCs did not appear to have gross defects in autophagy in the liver, heart, and macrophages [65]. There, the role of TPC2 in autophagy may be dependent on the conditions. Interestingly, Cang et al. suggested that ATP/mTOR phosphorylates and inhibits TPCs, thereby acting as a nutrient sensor to detect nutrient status in response to intracellular ATP and mTOR levels [65]. In contrast, in skeletal muscle, the loss of TPC2 leads to a reduced mTOR [135]. It seems that TPC2 and mTOR form a feedback regulatory loop in response to nutrient status.

### 4.3. Other Channels in Autophagy

Although most voltage gated Ca^2+^ channels (VGCCs) are found in the plasma membrane of excitable cells, P/Q-type VGCCs are recently reported to be expressed in lysosomes of both mice and fruit flies. Loss of VGCC leads to defects in autophagosome–lysosome fusion, indicating an important role of Ca^2+^ flux through this channel in autophagy [64].

## 5. Lysosomal Ca^2+^, Autophagy, and Cancer

An increasing number of tumorigenic pathways have been associated with an altered expression level or abnormal activation of Ca^2+^ regulatory membrane proteins including Ca^2+^ channels, transporters, or Ca^2+^-ATPases [136,137,138,139,140,141]. Abnormal autophagy has also been implicated in cancer development. It protects against the initiation of carcinogenesis, but also has a role enabling the survival of cells in solid tumors where nutrients are limited [142,143,144,145,146]. Given that lysosomal Ca^2+^ channels play an important role in autophagy, the role of lysosomal Ca^2+^ channels in cancer development has attracted great attention in recent years [24,25,147,148,149]. It is believed that impaired lysosomal Ca^2+^ signaling is a culprit in malignant tumor development [122]. Indeed, emerging evidence has demonstrated that lysosomal Ca^2+^ signaling underlies several cancer hallmarks involving proliferation, metastasis, and angiogenesis and contributes to multidrug resistance in cancer therapy [122,150,151,152,153,154,155,156,157,158,159,160]. Here we discuss the roles of the two major Ca^2+^ permeable channels, TRPMLs and TPCs, in cancer.

### 5.1. TRPMLs in Cancer

Recently, three groups have suggested independently that TRPML1 is required for tumor progression. Jung et al. [151] showed that TRPML1 expression was significantly elevated in HRAS-positive tumors and inversely correlated with patient prognosis. TRPML1 knockdown or inhibition selectively reduced the proliferation of cancer cells that express oncogenic but not wild-type HRAS. Mechanistically, they suggested that TRPML1 promotes cancer development by mediating cholesterol de-esterification and transport to maintain oncogenic HRAS in signaling-competent nanoclusters at the plasma membrane. In the meantime, Xu et al. [155] reported that in triple-negative breast cancer (TNBC), the expression level of TRPML1 was upregulated, promoting tumor proliferation via activating mTORC1 pathway. This is in agreement with previous findings that Ca^2+^ passing through TRPML1 is required for mTORC1 activation and cell growth [22,23]. In TNBC [155], TRPML1 also promoted lysosomal exocytosis [161,162,163,164], releasing lysosomal ATP [68,163,165] to the tumor microenvironment to facilitate cancer cell migration and invasion. Knockdown or inhibition of TRPML1 dampened tumor growth, cell migration, and invasion. Inversely, Kasitinon et al. [156] suggested that TRPML1 negatively regulated mTORC1 signaling to preferentially promote the survival and proliferation of melanoma cells, potentially by sustaining macropinocytosis and avoiding proteotoxic stress. Therefore, TRPML1 is necessary for cancer development by regulating multifaceted cellular signaling pathways. In line with this, TRPML1 inhibitor ML-SI1 has successfully suppressed the development of TNBC [155] and cancers bearing *HRAS* mutations [151]. Future studies should focus on optimizing known TRPML1 inhibitors (Table 1) and developing new drugs that specifically target on TRPML1 to treat some cancers.

Notably, the clinical course of patients with cancer is also related to the TRPML1 expression level. Elevated TRPML1 expression level is reported to associate with the poor prognosis of pancreatic ductal adenocarcinoma (PDAC) patients. Overall survival rate and recurrence-free survival are significantly lower in patients with high TRPML1 expression as compared with patients with low TRPML1 expression [166].

Although TRPML1 is often upregulated in cancer cells to promote cancer generation in glioblastoma TRPML1 agonist, MK6-83, reduce cell viability, and promote caspase-3-dependent apoptosis. Blocking TRPML1 dependent Ca^2+^ release or silencing TRPML1 abrogated these effects. Loss or reduction of TRPML1 transcripts strongly correlates with short survival in glioblastoma patients, suggesting that the reduction of TRPML1 expression may be negatively linked to prognosis for glioblastoma patients [167]. Therefore, the role of TRPML1 in cancer development is dependent on the types of cancers.

TRPML2 has been linked to glioma, because a high level of TRPML2 was detected in glioma tissues, and because its expression level increased with pathological grades [158]. Mechanistically, TRPML2 promoted tumor cell viability by inhibiting caspase-3 activity and increased proliferation through AKT and ERK1/2 phosphorylation. Silencing and knock-down of TRPML2 reduced proliferation of tumor cells and induced apoptosis [158].

The role of TRPML3 in cancer development has not been well studied. Wu et al. [168] reported that TRPML3 level is significantly downregulated in pancreatic adenocarcinoma tissues compared with non-tumor tissues. However, in squamous cell carcinoma and hepatocellular carcinoma, TRPML3 expression level was reported to be upregulated [169]. Therefore, further studies are needed to clarify the role of TRPML3 in different cancer types.

### 5.2. TPCs in Cancer

Emerging evidence has also suggested a role of TPCs in cancer development. Early studies suggested that TPC2 expression is increased in oral squamous cell carcinoma cell lines [170]. Later studies further suggested a link between TPC2 and melanoma [171,172], bladder cancer, leukemia, hepatocellular carcinoma [173], and breast cancer [174,175]. Suppressing TPC2 by siRNA or inhibitors reduced cancer cell migration and adhesion in vitro and decreased lung metastases of cancer cells in vivo [173,176]. Mechanistically, TPC2 may regulate autophagy [175] and β1-integrin recycling [173] to affect cancer progression. Interestingly, growing evidence has suggested that vascular endothelial growth factor (VEGF), the angiogenic factor, plays an important role in cancer progression by regulating vascularization. By using a murine model of VEGF-secreting melanoma, application of ned-19, an NAADP inhibitor, strongly inhibits tumor vascularization, growth, and metastases. At cellular level, ned-19 inhibits cell viability, proliferation, and migration; at molecular level, ned-19 suppresses VEGFR2 expression and VEGF-mediated lysosomal Ca^2+^ release in melanoma cells [176]. In agreement with the role of VEGF in angiogenesis, other studies suggest that in endothelial cells, VEGF induces lysosomal Ca^2+^ release through NAADP/TPC2 to regulate angiogenesis [177,178]. Altogether, these data suggest that VEGF promotes cancer progression by activating VEGFR2/NAADP/TPC2-mediated Ca^2+^ signaling pathway in both tumor cells and endothelial cells.

An earlier study showed that TPC1 transcripts are significantly higher than TPC2 transcripts in the SKBR3 human breast cancer cell line, highlighting the link between TPC1 expression and tumorigenicity [62]. Indeed, a recent study has suggested that NAADP-mediated TPC1 lysosomal Ca^2+^ release mobilizes ER Ca^2+^ via IP3R, subsequently promoting the proliferation of metastatic colorectal cancer (mCRC) cells by activating ERK and the PI3K/AKT signaling pathways [179].

## 6. Conclusions

It becomes evident that the lysosome acts as an important intracellular Ca^2+^ store. Given that Ca^2+^ signaling participates in processes that are important in cancer progression, such as autophagy, cell proliferation, growth and invasiveness, the tumor microenvironment, and resistance to anticancer therapies, it is therefore not surprising that exploration of the molecular mechanism underlying lysosomal Ca^2+^ homeostasis could be of great help in expanding our knowledge of the role of Ca^2+^ homeostasis in cancer development. In line with this, emerging evidence has suggested that Ca^2+^ release through lysosomal ion channels, the major lysosomal Ca^2+^ channels TRPML1 and TPC2, has been implicated in the progression of numerous cancers by controlling the autophagy pathway. Though more studies are needed to build a clear relation between lysosomal Ca^2+^ and cancer development and to underpin the precise mechanisms underlying the role of lysosomal Ca^2+^ in different types of cancers, developing potent and specific compounds targeting TRPML1 [151,153] and TPC2 [61] could be a promising therapeutic strategy to treat some cancers.

## Abbreviations

ML-SA: mucolipin synthetic agonist; ML-SI: mucolipin synthetic inhibitor; LyNa-VA: lysosomal Na^+^ channel voltage-dependent activator; LyNA: lysosomal Na^+^ channel agonist.
